# FRAS1-related extracellular matrix 3 (*FREM3*) single-nucleotide polymorphism effects on gene expression, amygdala reactivity and perceptual processing speed: An accelerated aging pathway of depression risk

**DOI:** 10.3389/fpsyg.2015.01377

**Published:** 2015-09-16

**Authors:** Yuliya S. Nikolova, Swetha P. Iruku, Chien-Wei Lin, Emily Drabant Conley, Rachel Puralewski, Beverly French, Ahmad R. Hariri, Etienne Sibille

**Affiliations:** ^1^Campbell Family Mental Health Research Institute of CAMHToronto, ON, Canada; ^2^Laboratory of NeuroGenetics, Department of Psychology & Neuroscience, Duke UniversityDurham, NC, USA; ^3^Department of Biostatistics, Graduate School of Public Health, University of PittsburghPittsburgh, PA, USA; ^4^23andMeMountain View, CA, USA; ^5^Department of Psychiatry, University of PittsburghPittsburgh, PA, USA; ^6^Department of Psychiatry, Department of Pharmacology and Toxicology, University of TorontoToronto, ON, Canada

**Keywords:** depression, aging, amygdala, Frem3, processing speed

## Abstract

The A allele of the FRAS1-related extracellular matrix protein 3 (*FREM3*) rs7676614 single nucleotide polymorphism (SNP) was linked to major depressive disorder (MDD) in an early genome-wide association study (GWAS), and to symptoms of psychomotor retardation in a follow-up investigation. In line with significant overlap between age- and depression-related molecular pathways, parallel work has shown that *FREM3* expression in postmortem human brain decreases with age. Here, we probe the effect of rs7676614 on amygdala reactivity and perceptual processing speed, both of which are altered in depression and aging. Amygdala reactivity was assessed using a face-matching BOLD fMRI paradigm in 365 Caucasian participants in the Duke Neurogenetics Study (DNS) (192 women, mean age 19.7 ± 1.2). Perceptual processing speed was indexed by reaction times in the same task and the Trail Making Test (TMT). The effect of rs7676614 on *FREM3* mRNA brain expression levels was probed in a postmortem cohort of 169 Caucasian individuals (44 women, mean age 50.8 ± 14.9). The A allele of rs7676614 was associated with blunted amygdala reactivity to faces, slower reaction times in the face-matching condition (*p* < 0.04), as well as marginally slower performance on TMT Part B (*p* = 0.056). In the postmortem cohort, the T allele of rs6537170 (proxy for the rs7676614 A allele), was associated with trend-level reductions in gene expression in Brodmann areas 11 and 47 (*p* = 0.066), reminiscent of patterns characteristic of older age. The low-expressing allele of another *FREM3* SNP (rs1391187) was similarly associated with reduced amygdala reactivity and slower TMT Part B speed, in addition to reduced BA47 activity and extraversion (*p* < 0.05). Together, these results suggest common genetic variation associated with reduced *FREM3* expression may confer risk for a subtype of depression characterized by reduced reactivity to environmental stimuli and slower perceptual processing speed, possibly suggestive of accelerated aging.

## Introduction

Despite increasing sample sizes, case-control genome-wide association studies (GWAS) of major depressive disorder (MDD) have yielded inconclusive results, with few single nucleotide polymorphisms (SNPs) surviving the commonly accepted genome-wide significance threshold of *p* = 5 × 10^−8^ and even fewer withstanding the test of a full replication in an independent sample (Wray et al., 2012; Major Depressive Disorder Working Group of the Psychiatric GWAS Consortium et al., 2013). The relative lack of success of these studies has been at least partially attributed to the heterogeneity inherent to the MDD diagnostic category (Major Depressive Disorder Working Group of the Psychiatric GWAS Consortium et al., 2013). Yet, despite the recognition in the literature of different MDD subtypes with presumably different pathophysiologies (Kessing, 2007), few genetic association studies take this heterogeneity into account, and those that do, have been inadequately powered to detect subtype-specific associations (Major Depressive Disorder Working Group of the Psychiatric GWAS Consortium et al., 2013).

Despite these initial set-backs, several follow-up investigations have resulted in partial replications or have linked suggestive SNP “hits” to specific symptom dimensions or biological endophenotypes, though not necessarily to the overall syndrome (Sullivan et al., 2009; Rietschel et al., 2010; Kohli et al., 2011; Kuehner et al., 2011; Li et al., 2013; Schuhmacher et al., 2013). In light of the immense diagnostic, and presumably biological heterogeneity of MDD, such partial replications and mechanistic follow-up studies may in fact inform an improved understanding of the etiology of specific subtypes of MDD as well as help facilitate the delineation of distinct biological pathways of risk.

A SNP (rs7676614) within the human FRAS1-related extracellular matrix protein 3, encoded by the *FREM3* gene, was among the top hits in an early GWAS of MDD, where the major A allele was associated with nominally higher risk relative to the minor G allele (*p* = 9.52 × 10^−5^ meta-analyzed across two independent samples) (Muglia et al., 2010). Importantly, while this SNP was not associated with MDD diagnosis in a follow-up study, it was specifically and strongly linked to symptoms of psychomotor retardation (Shi et al., 2012). Psychomotor symptoms in depression can range from retardation to agitation and have been proposed to constitute an important source of disorder heterogeneity (Sobin and Sackeim, 1997; Leventhal et al., 2008; Schrijvers et al., 2008). Thus, the association between rs7676614 and symptoms of retardation may not only explain the lack of a stronger link between the SNP and overall MDD diagnosis, but also offer mechanistic clues as to the specific biological pathway through which this SNP may increase MDD risk.

A largely independent line of research drawing on human postmortem gene expression data has demonstrated a significant overlap between molecular pathways implicated in depression and normal aging (Wolkowitz et al., 2010; Sibille, 2013). Specifically, it has been proposed that MDD or risk thereof may stem at least partially from molecular processes resulting in gene expression patterns reminiscent of premature, or accelerated, aging of the brain (Douillard-Guilloux et al., 2013; Sibille, 2013). Intriguingly, *FREM3* is among the genes whose expression declines as a function of normal aging (Glorioso et al., 2011). This steady decline has been shown to occur in the amygdala and the anterior cingulate cortex (ACC), both of which have been extensively implicated in the pathophysiology of depression (Davidson et al., 2002). While this prior work has used a relatively small cohort enriched for individuals over the age of 50, thus possibly not fully capturing age-related changes in expression pattern occurring throughout the entire adult lifespan, it strongly suggests *FREM3* may contribute to age-related pathways of depression risk. Furthermore, the retardation of psychomotor function, in addition to being a symptom of a subtype of depression, is also a concomitant of older age (Seidler et al., 2010). Taken together, these data raise the intriguing possibility that the A allele of the *FREM3* rs7676614 polymorphism may predispose to depression via an accelerated brain aging pathway, or, alternatively, the minor G allele may be associated with relative resilience to, or slowing of, the normal changes associated with aging.

Depression and aging are both associated with profound changes in cognitive and affective processing in the brain (Milham et al., 2002; Whalen et al., 2002; Fitzgerald et al., 2008; Disner et al., 2011; Kehoe et al., 2013; Kennedy et al., 2015). A large body of work utilizing functional magnetic resonance imaging (fMRI) in patients with MDD has revealed dysregulation within a corticolimbic circuitry involved in the perception and regulation of emotion and affect, within which the amygdala serves as a hub. The majority of these studies demonstrate relative amygdala hyperactivity in response to negative stimuli (Whalen et al., 2002; Victor et al., 2010; Yang et al., 2010). However, a notable minority of studies has associated depression (Thomas et al., 2001) or depression risk (Wolfensberger et al., 2008) with relatively blunted amygdala response to threat, suggesting there may be a depression subtype associated with reduced reactivity to environmental stimuli. Notably, amygdala reactivity is also reduced in healthy older adults (Fischer et al., 2005, 2010; Tessitore et al., 2005). Consistent with molecular pathology findings in depression (Douillard-Guilloux et al., 2013), it is possible that a premature forward shift along this normal age-related trajectory of corticolimbic function may contribute to risk of depression or one of its subtypes. Conversely, factors that slow progression along this trajectory may confer resilience.

In the current study, we leveraged data from the ongoing Duke Neurogenetics Study (DNS) and a postmortem cohort, to test the hypothesis that rs7676614 would modulate risk of depression via accelerating normal age-related changes in neuropsychological function and gene expression patterns. Specifically, we hypothesized that, regardless of current depressive symptomatology, the A allele would bias amygdala reactivity, perceptual processing speed, and gene expression toward a pattern reminiscent of older age. We further sought to confirm the effect of age on *FREM3* gene expression in the current postmortem cohort, which is better powered to detect trajectories of gene expression change across the entire adult lifespan than previous ones (Erraji-Benchekroun et al., 2005; Glorioso et al., 2011), while also extending the results to the orbitofrontal and ventrolateral prefrontal cortices (Brodmann areas 11 and 47). Finally, we explored the possible involvement of *FREM3* in depression risk by linking additional functional variation in the same gene (rs1391187) to behaviorally relevant differences in *in vivo* brain function.

## Methods

### Participants

Neuroimaging and genetic data were derived from 365 non-Hispanic Caucasian participants (192 women, mean age 19.76 ± 1.23) who had successfully completed the ongoing Duke Neurogenetics Study (DNS) and whose BOLD fMRI data survived a stringent multi-level quality control procedure (Supplementary Methods). The DNS assesses a range of behavioral and biological traits among young adult, university students. All participants provided informed consent in accordance with Duke University guidelines, and were in good general health. All participants were free of the following exclusionary criteria, as determined by self-report: (1) medical diagnoses of cancer, stroke, diabetes requiring insulin treatment, chronic kidney or liver disease, or lifetime history of psychotic symptoms; (2) use of psychotropic, glucocorticoid, or hypolipidemic medication; and (3) conditions affecting cerebral blood flow and metabolism (e.g., hypertension). Participants were also screened for DSM-IV Axis I and select Axis II diagnoses (Antisocial and Borderline Personality Disorder) using the eMINI (Sheehan et al., 1998). Current or lifetime diagnosis of a disorder was not exclusionary. However, all participants were free of psychotropic medication, including recreational drugs, at the time of study participation. Notably, controlling for current or past DSM-IV diagnosis did not significantly alter our results. Thus, we report results from models not including diagnosis as a covariate. Detailed diagnostic information can be found in Supplementary Table 1.

### BOLD fMRI paradigm

Our amygdala reactivity paradigm has been described in detail previously (Carré et al., 2013). Briefly, the amygdala reactivity paradigm consists of 4 blocks of a face-processing task interleaved with 5 blocks of a sensorimotor control task. During task blocks, participants are simultaneously presented with three faces (with neutral, angry, fearful, or surprised expressions) and are asked to indicate which one of two faces shown in the bottom of the screen is identical to a target face shown on top. During control blocks, participants perform an analogous task with simple geometric shapes. Here, we focused on amygdala reactivity in the contrast of face blocks vs. control blocks (i.e., All Faces > Shapes). To ensure greater convergence between our *in vivo* neuroimaging and our post-mortem data analyses, we further probed the effect of genotype on activity in BA11/47, corresponding to regions of the orbitofrontal cortex (OFC) and ventrolateral prefrontal cortex (vlPFC). In a follow-up exploratory analysis we probed genotype effects on threat-specific amygdala reactivity in the Anger + Fear > Shapes condition.

### Perceptual processing speed

Perceptual processing speed was assessed using reaction times (RT) recorded during the face and shape-matching task. Notably, even though the stimuli remained on the screen for a fixed amount of time to ensure uniform perceptual exposure, participants were instructed to match the stimuli as quickly as they can, while maintaining accuracy. RTs were recorded in all trials. Accuracy was close to ceiling throughout the task, but was slightly higher in the Faces (98.7% ± 0.05), relative to the Shapes (98.0% ± 0.05) condition (*p* < 0.001). Only RTs from correct trials in both conditions were used in analyses.

To further probe perceptual and cognitive processing speed we used the Trail Making Test (TMT, Bowie and Harvey, 2006). This test is designed to measure processing speed, cognitive flexibility as well as visuomotor skills. Part A of the TMT (Trails A) asks participants to connect encircled numbers 1 through 25 sequentially on a piece of paper. In Part B of the TMT (Trails B) participants connect encircled numbers and letters, in numerical and alphabetical order, respectively, while alternating between numbers and letters. Numbers and letters are distributed on the paper in a semi-random fashion that allows them to be connected without drawing overlapping lines. The primary outcome of interest in both Parts A and B of this test is the overall time it takes for participants to complete the task. Trails A is believed to gauge visual search and motor speed skills, while Trails B is believed to tap into higher-level cognitive skills, such as mental flexibility. Outliers greater than 60 s on Trails A (*n* = 2) or 90 s (*n* = 3) on Trails B were removed from analyses. Number of errors was controlled for alongside gender and age in all analyses involving the TMT.

### Self-report and behavioral measures

Recent and early life stress were assessed using a modified version of the Life Events Scale for Students (LESS, Nikolova et al., 2012) and the Childhood Trauma Questionnaire (CTQ, Bernstein et al., 2002), respectively. Current depression and anxiety were assessed using the Center for Epidemiological Studies-Depression (CES-D, Radloff, 1977) scale and the short form of the Mood and Anxiety Symptom Questionnaire (MASQ, Watson et al., 1995). To assess the effects of genotype on personality traits which may predispose to or protect against depression, we analyzed response data on the NEO personality inventory (Costa and McCrae, 1992). We focused specifically on the neuroticism (NEON) and extraversion (NEOE) factors, which have been most extensively mapped onto specific neurobiological substrates (Wright et al., 2006; Aghajani et al., 2014), as well as differential risk or resilience for psychopathology (Campbell-Sills et al., 2006; Bienvenu et al., 2007; Lamers et al., 2012). The Wechsler Abbreviated Scale of Intelligence™ (WASI™, Wechsler, 1999) was administered to assess general intelligence, which was used as a covariate in analyses involving the TMT.

### Genotyping

Genotyping of the *in vivo* cohort was facilitated through 23andMe (23andMe, Inc., Mountain View, CA). Genomic DNA from all participants was isolated from buccal cells and leukocytes derived from Oragene DNA self-collection kits (DNA Genotek, Inc., Kanata, Ontario, Canada) customized for 23andMe. DNA extraction and genotyping were performed at the National Genetics Institute (NGI), a CLIA-certified clinical laboratory and subsidiary of Laboratory Corporation of America. The Illumina Omni Express Plus chip (Illumina, Inc., San Diego, CA, USA) and a custom array containing an additional ~300,000 SNPs were used to provide genome-wide data (Eriksson et al., 2010; Do et al., 2011; Tung et al., 2011). The Illumina Omni Express Plus chip included the rs7676614 SNP. In our final sample, genotype frequencies for rs7676614 did not deviate from Hardy-Weinberg equilibrium (169 A homozygotes, 166 AG heterozygotes, 30 G homozygotes, χ^2^ = 1.49; *p* = 0.22). Furthermore, the allele frequencies in the current sample (A allele: 0.31, G allele: 0.69) were similar to those reported for populations of European ancestry (current sample: A allele frequency 0.31, G allele: 0.69; reference sample from 1000 Genomes, A allele: 0.35, G allele: 0.65).

### Postmortem gene expression

The postmortem cohort has been described in detail previously (Seney et al., 2013). Briefly, data were derived from 211 individuals (44 women, mean age 50.76 ± 14.91, range 16–96) with no history of neuropsychiatric illness. After consent from next-of-kin was obtained, postmortem brains were collected in the Allegheny County Coroner's Office (Pittsburgh, PA) using procedures approved by University of Pittsburgh's Institutional Review Board and Committee for Oversight of Research Involving the Dead. The absence of psychiatric DSM-IV diagnosis was determined based on psychopathology, medical and social histories, as well as history of substance abuse. Individuals were also screened for the absence of neurodegenerative disorders by neuro-pathological examination.

Total RNA was extracted from frozen samples using TRIzol® (Invitrogen Life Technologies, Carlsbad, CA, USA) following manufacturer's protocol (see Supplementary Methods). Technical parameters of all RNA samples were within the range of desired RNA quality for large-scale gene expression studies (RNA integrity number: 8.02 ± 0.74, RNA ratio: 1.53 ± 0.35). RNA samples were processed for microarray by the Gene Expression & Genotyping Core Facility at Case Western Reserve University. Briefly, with 150 ng of total RNA, cDNA was synthesized by Ovation PicoSL WTA System V2 and labeled with Encore Biotin Module (both from NuGEN Technologies, San Carlos, CA). 2.5 μg of cDNA was hybridized on Affymetrix® Human Gene 1.1 ST arrays (Affymetrix, Santa Clara, CA), covering over 30,000 coding transcripts. Array hybridization, washing, and staining were conducted on GeneTitan® (Affymetrix) according to the manufacturer's protocol. Gene expression values were extracted via Expression Console build 1.2.1.20 using RMA method and quantile-normalization to eliminate batch effects. Gene expression probes were processed in gene-level and converted to log2 scale to perform further analysis. One coding transcript corresponding to the *FREM3* gene was represented on the array. Analyses were restricted to Caucasian individuals, whose samples passed quality control (*n* = 169, 44 women, mean age 50.8 ± 14.9; see also Supplementary Methods).

Total DNA was extracted from fresh frozen brain samples with Qiagen DNA mini kit following manufacturer's protocol. Genotype calls were generated using Affymetrix Genotyping Console version 4.1.3 by a Birdseed v2 algorithm, which uses EM to drive maximum likelihood fit of two dimensional Gaussian mixture models. As the *FREM3* rs7676614 SNP was not available on the Affymetrix SNP Array 6.0, a perfect proxy SNP, rs6537170 (*R*^2^ = 1.00, D′ = 1.00), was identified using data from the 1000 Genomes project (Genomes Project et al., 2012) accessed via the online SNAP Proxy Search Tool of the Broad Institute (Johnson et al., 2008). This proxy SNP was used in all analyses involving postmortem samples. We probed the effect of rs6537170 on *FREM3* mRNA levels in samples derived from both BA11 and BA47 in 169 Caucasian individuals. An additional 57 SNPs within or near the *FREM3* gene (50 kb up- or downstream) were examined for association with *FREM3* expression (Supplementary Table 2). To further explore the involvement of *FREM3* in depression-related phenotypes, we selected the SNP with the strongest impact on *FREM3* expression (rs1391187) and evaluated its effect (via proxy SNP rs1909022, *R*^2^ = 1.00, D′ = 1.00, 1000 Genomes) on the same *in vivo* neural and behavioral phenotypes tested for rs7676614.

### Statistical analyses

Three sets of models were tested for all *in vivo* phenotypic associations: additive, minor allele dominant, and minor allele recessive. In the additive model, linear regressions using number of major alleles as a predictor of amygdala reactivity or perceptual processing time were conducted in IBM SPSS Statistics 21 (IBM, Armonk, NY). In the dominant model, ANOVAs using genotype (minor allele carrier vs. major allele homozygote, or major allele carrier vs. minor allele homozygote) predicting outcomes were used. In the postmortem cohort, a general power function model was used to assess the effects of age on gene expression scores residualized for potential confounding covariates (e.g., postmortem interval, pH, RNA integrity number, race, and gender). This model was adopted in order to allow for the detection of non-linear age-related trajectories. The effects of genotype on expression was tested using *FREM3* expression scores residualized for those potential confounds in addition to estimated non-linear age effect, ethnic background (Price et al., 2006), and 16 principal components derived from transcriptome-wide analysis of gene expression data (Liang et al., 2013). Mediation analyses in the *in vivo* cohort were conducted using Models 4 and 6 of the PROCESS macro in SPSS (Hayes, 2013). Bootstrapped bias-corrected confidence intervals (CIs) for each indirect effect were generated using 5000 bootstrapping iterations.

## Results

### Main effect of task and demographics

**Figure 1:**
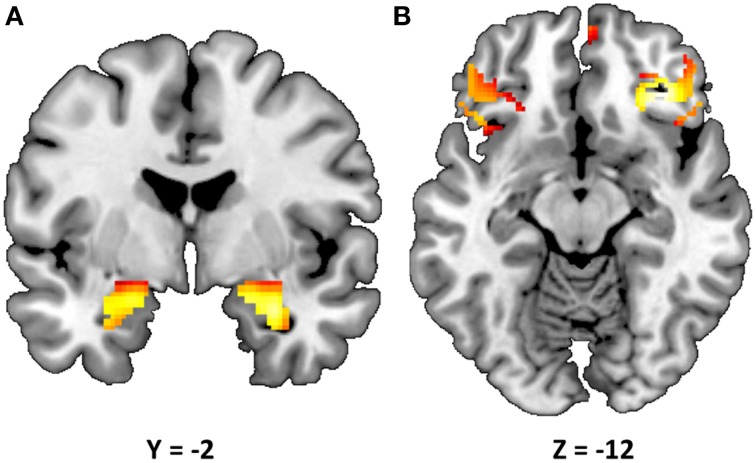
**Statistical parametric map illustrating mean bilateral activity for the Faces > Shapes contrast**. Activation clusters are presented for **(A)** the amygdala (peak voxel activation in the left hemisphere: *x* = −22, *y* = −6, *z* = −18, *t* = 28.55, *p* < 0.000001, *kE* = 179; right hemisphere: *x* = 28, *y* = −4, *z* = −20, *t* = 32.82, *p* < 0.000001, *kE* = 199) and **(B)** Brodmann areas 11 and 47 (peak voxel activation in the left hemisphere: *x* = 58, *y* = 28, *z* = 0, *t* = 15.60, *p* < 0.000001, *kE* = 143; right hemisphere: *x* = −52, *y* = 40, *z* = −2, *t* = 14.47, *p* < 0.000001, *kE* = 24). The clusters are overlaid onto canonical structural brain images in the coronal plane (*y* = −2) and axial planes (*z* = −12), respectively.

Consistent with prior research (Nikolova and Hariri, 2012; Swartz et al., 2015), our task resulted in significant amygdala reactivity bilaterally for the Faces > Shapes contrast (Figure 1A). Further analyses revealed significant activity in BA11 and BA47 (Figure 1B). Partially confirming prior work (Nikolova et al., 2012), we found that men had greater amygdala reactivity than women in the left [*t*_(363)_ = 2.54, *p* = 0.012], but not the right hemisphere [*t*_(363)_ = 1.30, *p* = 0.193]. When gender was accounted for, amygdala reactivity was further modulated by age, such that older participants showed a trend towards lower amygdala reactivity (left hemisphere: *b* = −0.087, *p* = 0.096; right hemisphere: *b* = −0.090, *p* = 0.085). There were no differences in age, gender composition and estimated IQ among genotype groups (Table 1). However, in light of the effects of gender and age on amygdala reactivity, all analyses were conducted with and without gender and age as covariates.

**Table 1:** **Summary of demographic characteristics and estimated IQ represented as a function of *FREM3* rs7676614 genotype**.

	***FREM3* rs7676614**			***p***	***p* (additive)**
	**GG**	**AG**	**AA**		
*n*	30	166	169	‘	‘
Male/Female	14/16	76/90	83/86	0.827	‘
Age (mean ± SD)	19.83 ± 1.26	19.83 ± 1.25	19.67 ± 1.20	0.499	0.278
Estimated IQ	125.33 ± 6.47	123.70 ± 7.12	123.26 ± 7.70	0.358	0.200
CTQ	31.13 ± 6.64	31.09 ± 6.70	31.85 ± 8.23	0.632	0.390
LESS	3.97 ± 2.39	3.98 ± 2.74	4.49 ± 3.26	0.270	0.138
NEO-N	77.83 ± 23.64	82.58 ± 20.59	82.50 ± 24.10	0.549	0.490
NEO-E	125.30 ± 20.08	120.81 ± 21.40	122.09 ± 20.92	0.543	0.839

*The table includes p-values from both a mean comparison (ANOVA) and an additive (regression) model*.

### Genetic effects on amygdala reactivity

**Figure 2:**
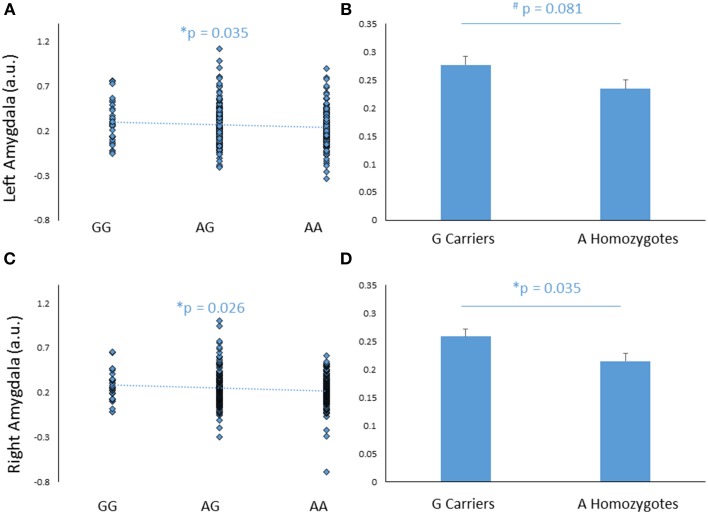
**Effects of *FREM3* rs7676614 genotype on amygdala reactivity**. Increasing number of A alleles was associated with decreased amygdala reactivity in the left **(A)** and right **(C)** hemisphere. In a G allele dominant model, A homozygotes showed a trend toward decreased amygdala reactivity in the left hemisphere **(B)** and significantly decreased amygdala reactivity than G carriers in the right hemisphere **(D)**. Error bars represent standard error of the mean. ^*^p < 0.05, ^#^*p* < 0.10, a.u. = arbitrary units.

*FREM3* rs7676614 genotype modulated amygdala response, such that increasing number of major (A) alleles was associated with significantly decreased amygdala reactivity in the right (*b* = −0.109, *p* = 0.038), and marginally decreased reactivity in the left (*b* = −0.101, *p* = 0.055) hemisphere (Figures 2A,C). The effect was significant bilaterally when covarying for gender and age (right hemisphere: *b* = −0.116, *p* = 0.026; left hemisphere: *b* = −0.110, *p* = 0.035). When a G allele dominant model was tested, individuals homozygous for the risk A allele were found to have relatively reduced amygdala reactivity for the All Faces > Shapes contrast, when compared to carriers of the G allele. The effect was stronger on the right side [left hemisphere: *F*_(1, 363)_ = 3.06, *p* = 0.081; right hemisphere: *F*_(1, 363)_ = 4.46, *p* = 0.035; Figures 2B,D]. The significance of these results did not change when gender and age were controlled for [left hemisphere: *F*_(1, 361)_ = 3.85, *p* = 0.051; right hemisphere: *F*_(1, 361)_ = 5.20, *p* = 0.023]. Notably, no significant effect of genotype emerged in threat-specific contrasts (*p*>0.11), suggesting a broader hypo-reactivity of the amygdala to environmental salience rather than a threat-specific blunting of amygdala response. Consistent with this conceptualization, neither current, nor early life stress interacted with *FREM3* genotype to predict amygdala response (*p*>0.12). Importantly, there were no differences in self-reported early or recent stress between genotype groups (*p*>0.10).

An exploratory whole-brain analysis revealed no effect of *FREM3* rs7676614 genotype on activity in any other brain region. Further region of interest analyses revealed no effect on activity in BA11 and BA47 (*p*>0.10).

### Genetic effects on processing speed

#### Reaction times

**Figure 3:**
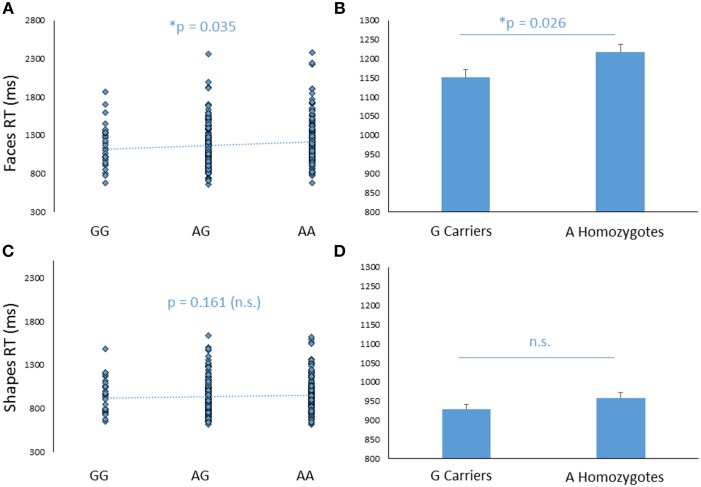
**Effects of *FREM3* rs7676614 genotype on reaction times in the perceptual face matching task**. Increasing number of A alleles was associated with slower reaction times in the Faces **(A)**, but not the Shapes **(C)** condition. These results were confirmed in a G allele dominant model, where A homozygotes showed slower reaction times than G carriers in the Faces **(B)** but not the Shapes condition **(D)**. Error bars represent standard error of the mean. ^*^p < 0.05, ^#^*p* < 0.10.

In addition to its effect on amygdala response, *FREM3* genotype was found to modulate processing speed as indexed by behavioral measures collected during the BOLD fMRI paradigm. Specifically, increasing number of A alleles was associated with slower RTs in the Faces (*b* = −0.107, *p* = 0.035; controlling for gender and age: *b* = −0.109, *p* = 0.032), but not in the Shapes (*p*>0.2; Figures 3A,C) condition. Similarly, G allele carriers had faster RTs relative to A homozygotes in the Faces [A homozygotes: 1.22 ± 0.28 s; G carriers: 1.15 ± 0.27 s; *F*_(1, 363)_ = 4.89, *p* = 0.028; controlling for gender and age: *F*_(1, 361)_ = 5.00, *p* = 0.026], but not in the Shapes blocks [A homozygotes: 0.96 ± 0.19 s; G carriers: 0.93 ± 0.19 s; *F*_(1, 363)_ = 1.97, *p* = 0.161; controlling for gender and age: *F*_(1, 361)_ = 2.22, *p* = 0.137; Figures 3B,D]. Notably, RTs were slower overall in the Faces, compared to the Shapes condition, possibly reflecting the relative perceptual complexity of the stimuli to be matched [Faces: 1.80 ± 0.28 s; Shapes: 0.94 ± 0.19 s *F*_(1, 361)_ = 0.826, *p* < 0.0001]. Thus, perhaps the lack of genotype effect on RT in the Shapes condition may reflect performance ceiling effects. There were no differences in accuracy between genotype groups (*p*>0.27).

#### Trail making test

**Figure 4:**
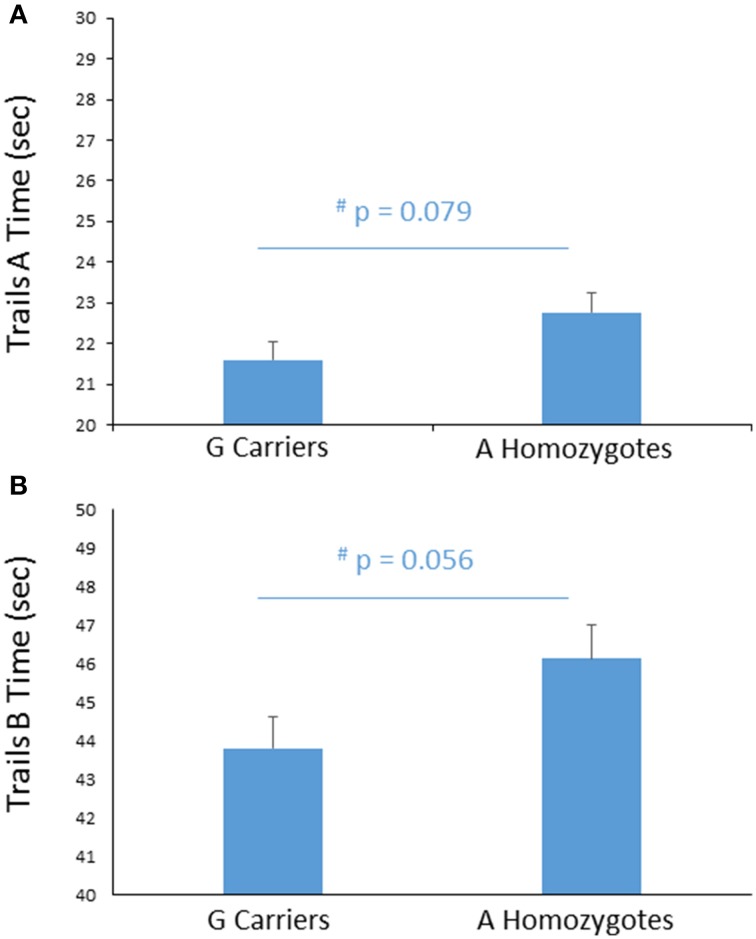
**Effects of *FREM3* rs7676614 genotype on TMT performance**. Relative to G carriers, A allele homozygotes showed a trend toward slower performance in both Part A **(A)** and Part B **(B)** of the TMT. Error bars represent standard error of the mean. ^*^p < 0.05, ^#^*p* < 0.10.

To further probe the effect of *FREM3* genotype on perceptual processing speed, we compared genotype groups on their performance on the TMT Parts A and B, administered outside the scanner. Despite A homozygotes' being nominally slower to complete Trails A, the difference in performance reached only trend levels [A homozygotes: 22.71 ± 6.60 s; G carriers: 21.64 ± 5.79 s, *F*_(1, 355)_ = 3.11, *p* = 0.079; Figure 4A]. Larger genotype differences emerged in Trails B performance, where A homozygotes were slower to complete the task by an average of 2.14 s [A homozygotes: 46.02 ± 12.73 s; G carriers: 43.89 ± 11.03 s, *F*_(1, 355)_ = 3.69, *p* = 0.056; Figure 4B]. No additive effect of number of A alleles was observed on either task (*p*>0.12). Notably, there were no genotype effects on number of errors in either Trails A or Trails B (*p*>0.39) and the differences in speed occurred in the absence of genotype differences in estimated IQ (Table 1). Furthermore, the effects remained trending when IQ was controlled for alongside gender and age (Trails A: *p* = 0.089; Trails B: *p* = 0.076).

There was no association between amygdala reactivity and processing speed, as reflected by either RT or TMT, either in the overall sample or for any specific genotype group (*p*>0.10).

### Postmortem gene expression

**Figure 5:**
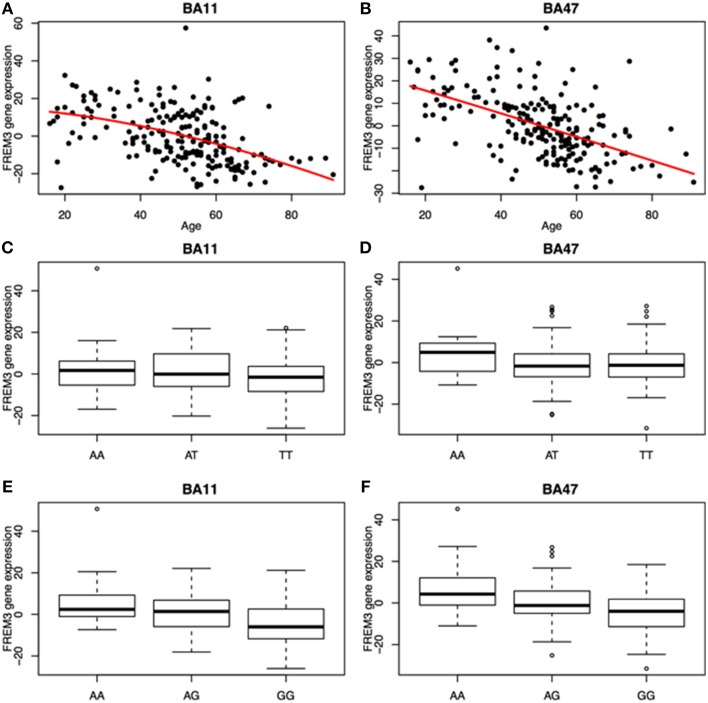
**Age was associated with a robust decrease in *FREM3* gene expression in both BA11 (A) and BA47 (B)**. In addition, the major T allele of rs6537170 (proxy for rs7676614), was associated with trend-level reductions in gene expression in BA11 **(C)** and BA47 **(D)**. Finally, the major G allele of rs1391187 was also associated with decreased gene expression in BA11 **(E)** and BA47 **(F)**. Each box in **(C–F)** represents the interquartile range of the data and the whiskers extend to the most extreme data point which is no more than 1.5 times the interquartile range from the box.

We next probed the effect of age and *FREM3* genotype on *FREM3* mRNA levels in human postmortem tissue. Age was associated with a robust decline in *FREM3* expression in both BA11 and BA47 following a near-linear trajectory (Figures 5A,B). In addition, our proxy SNP showed a trending effect, such that the major allele was associated with relatively reduced gene expression in both BA11 (*p* = 0.062; Figure 5C) and BA47 [Figure 5D, *p* = 0.08; *p* = 0.066 based on an adaptively-weighted (AW) meta-analysis (Li and Tseng, 2011) across both regions] in a pattern reminiscent of that seen in older age. Importantly, these results were adjusted for gender and estimated effects of chronological age at time of death.

### Additional FREM3 variants

**Figure 6:**
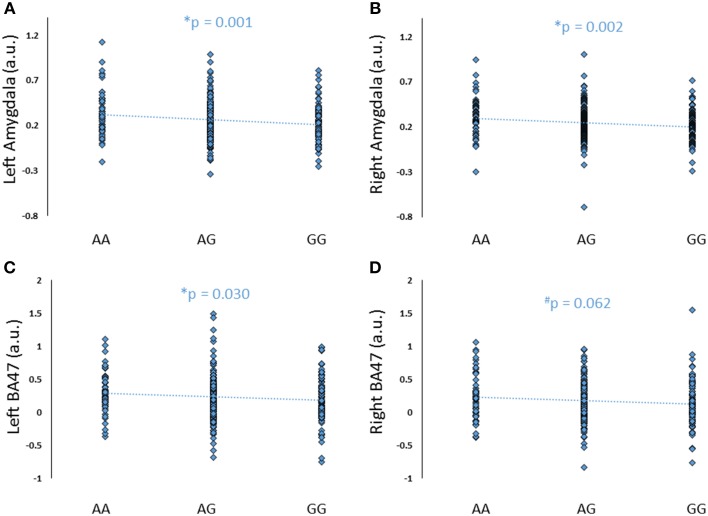
**Effects of *FREM3* rs1909022 genotype (proxy for rs1391187) on amygdala and BA47 activity (A–D)**. Increasing number of major (G) alleles was associated with reduced reactivity in both brain regions and hemispheres. ^*^p < 0.05, ^#^*p* < 0.10, a.u. = arbitrary units.

**Figure 7:**
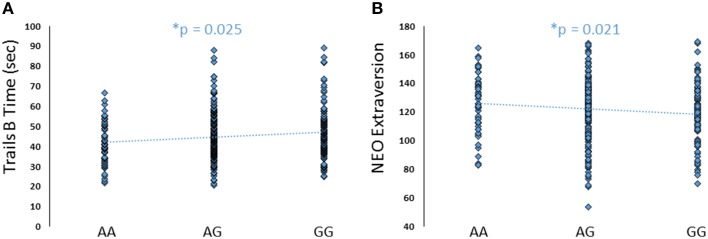
**Effects of *FREM3* rs1909022 genotype on TMT Part B performance and extraversion**. Increasing number of major (G) alleles was associated with relatively slower TMT performance **(A)** and lower levels of extraversion **(B)**. ^*^p < 0.05.

To further explore the potential involvement of the *FREM3* gene in regulating depression-relevant neural and behavioral phenotypes, we identified rs1391187 as the SNP most strongly modulating *FREM3* expression across both BA11 (*p* = 2.17 × 10^−5^, Figure 5E) and BA47 (*p* = 2.47 × 10^−6^, Figure 5F, Supplementary Table 2) in our postmortem cohort, and tested its effects (via proxy SNP rs1909022, *R*^2^ = 1.00, D′ = 1.00), on neural activity and perceptual processing speed in our *in vivo* cohort. In striking similarly to rs7676614, the lower-expressing major allele at rs1391187/rs1909022 was also associated with reduced amygdala reactivity bilaterally (left amygdala: *b* = −0.167; *p* = 0.001; right amygdala: *b* = −0.159; *p* = 0.002; Figures 6A,B) as well as slower speed in the TMT Part B (*b* = 0.114; *p* = 0.025, Figure 7A). These effects remained significant when controlling for gender and age (left amygdala: *b* = −0.170; *p* = 0.001, right amygdala: *b* = −0.162; *p* = 0.002; Trails B, adjusted for IQ: *b* = 0.111; *p* = 0.028). Notably, there were no significant differences in gender composition or estimated IQ among rs1909022 genotype groups (Table 2). While there was a main effect of age (*p* = 0.045), such that heterozygotes were slightly but significantly older than GG homozygotes (*p* = 0.024, LSD-corrected), there was no linear association between number of minor/major alleles and age (*p* = 0.486).

**Table 2:** **Summary of demographic characteristics and estimated IQ represented as a function of *FREM3* rs1909022 genotype**.

	***FREM3* rs1909022**			***p***	***p* (additive)**
	**GG**	**AG**	**AA**		
*n*	111	197	57	‘	‘
Male/Female	49/62	100/97	24/33	0.367	‘
Age (mean ± SD)	19.58 ± 1.18	19.90 ± 1.24	19.60 ± 1.24	**0.045**	0.486
Estimated IQ	123.12 ± 6.96	124 ± 7.51	123.37 ± 7.57	0.575	0.651
CTQ	31.67 ± 7.34	31.11 ± 7.49	32.18 ± 7.48	0.591	0.857
LESS	4.16 ± 2.97	4.23 ± 3.13	4.26 ± 2.43	0.974	0.821
NEO-N	84.19 ± 23.90	81.51 ± 21.67	80.40 ± 22.66	0.494	0.253
NEO-E	119.11 ± 19.55	121.67 ± 21.88	127.35 ± 20.23	0.055	**0.021**

*The table includes p-values from both a mean comparison (ANOVA) and an additive (regression) model. Significant values are highlighted in bold font*.

There was no effect of rs1391187/rs1909022 genotype on RT in the Faces or Shapes matching condition or the TMT Part A (*p*>0.20). Genotype groups did not differ on self-reported stress (*p*>0.30) and stress did not interact with rs1391187/rs1909022 genotype to predict amygdala reactivity or processing speed (*p*>0.10). Notably, rs1391187/rs1909022 and rs7676614 are not in high LD (*R*^2^ = 0.182, D′ = 0.481, 1000 Genomes) and did not interact with each other to predict any phenotype of interest (*p*>0.19), suggesting their contributions to differences in *FREM3* expression and function may be independent.

An exploratory whole-brain analysis revealed no effect of *FREM3* rs1391187/rs1909022 genotype on activity in any other brain region. However, ROI analyses revealed that the major allele was associated with significantly decreased activity in BA47 (Figures 6C,D).

### Link to self-report measures

**Figure 8:**
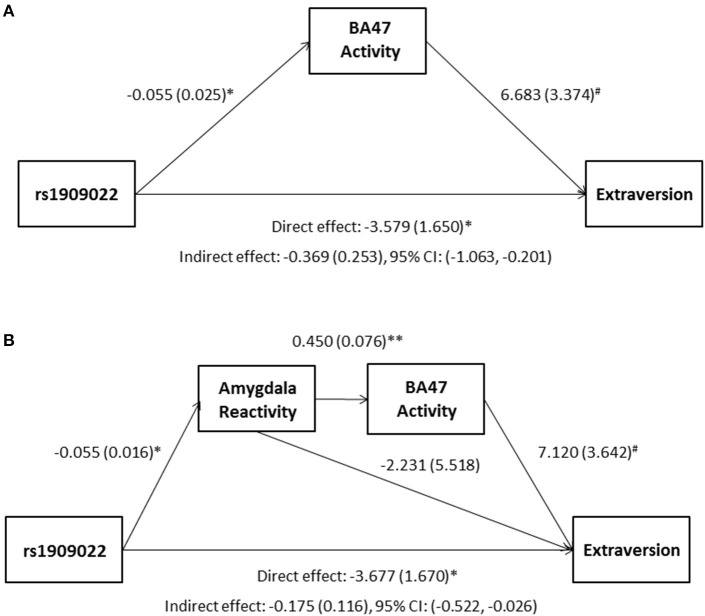
**Results from mediation models wherein activity in BA47 mediated the association between rs1909022 genotype and extraversion scores by itself (A) or in conjunction with amygdala reactivity (B)**. Raw regression coefficients are presented for each path, along with standard errors in parentheses. Bootstrapped 95% confidence intervals are presented for the indirect (mediation) effects. ^**^p < 0.005, ^*^p < 0.05, *#p* < 0.10. CI, Confidence interval.

Although there were no differences in measures of current mood and neuroticism between genotype groups as defined by either SNP (*p*>0.10), increasing number of major alleles at rs1391187/rs1909022 was associated with lower extraversion, as assessed by the NEOE (no covariates: *b* = −0.121, *p* = 0.021, with covariates; *b* = −0.124, *p* = 0.017, Figure 7B). This effect was driven primarily by the Activity subscale (no covariates: *b* = −0.143, *p* = 0.006; with covariates: *b* = −0.145, *p* = 0.005). Furthermore, NEOE was positively correlated with BA47 activity in the left hemisphere, such that BA47 activity negatively mediated the association between number of rs1391187/rs1909022 major alleles and NEOE scores (Figure 8A). Finally, there was a positive correlation between amygdala reactivity and BA47, such that when amygdala and BA47 activity were entered sequentially into a mediation model, they were both found to be significant mediators of the link between rs1391187/rs1909022 genotype and NEOE (Figure 8B). Importantly, a model where amygdala reactivity was entered as a mediator more proximal to extraversion did not result in significant mediation (*b* = 0.098, *SE* = 0.264, 95% CI: –0.366, 0.704).

## Discussion

In this study we show that the major A allele of the *FREM3* rs7676614 polymorphism is associated with blunted amygdala reactivity to socially salient stimuli, as well as reduced processing speed, as indexed by slower reaction time in a perceptual matching task and visuomotor performance in the Trails Making Test. These results are consistent with prior epidemiological and clinical studies associating the A allele with relatively heightened risk of depression (Muglia et al., 2010) as well as symptoms of psychomotor retardation (Shi et al., 2012). We also confirm and extend prior basic molecular work (Glorioso et al., 2011) by showing that *FREM3* expression in postmortem human prefrontal cortex (BA11 and BA47) tissue robustly decreases with age. Moreover, we show that the A allele of rs7676614 is associated with a trend toward reduced *FREM3* gene expression, shifting this molecular phenotype toward a pattern reminiscent of older age. Finally, we demonstrate convergent *in vivo* phenotypic effects of rs1391187, the SNP showing the strongest modulatory impact on *FREM3* expression in our postmortem cohort. Specifically, we show that the lower-expressing major allele of rs1391187 (assessed via proxy SNP rs1909022) is associated with relatively reduced amygdala reactivity, slower visuomotor performance in the TMT Part B, blunted activity in BA47, as well as reduced extraversion. Taken together, and in light of prior work demonstrating reduced amygdala reactivity and slower processing speeds in healthy older adults (Tessitore et al., 2005), our results suggest that the major alleles at both rs7676614 and rs1391187/rs1909022 may increase depression risk by precipitating the premature activation of biological pathways implicated in the normal molecular aging of the brain (Sibille, 2013). Conversely, the minor alleles at these loci may be associated with relative resilience to depression via a comparative slowing down of normal age-related processes.

The conceptual link between depression and accelerated aging (Wolkowitz et al., 2011; Sibille, 2013) may seem at odds with epidemiological data suggesting MDD is diagnosed less frequently in older, relative to young adults (Hasin et al., 2005). This seeming inconsistency may be due to the fact that late-life depression has a unique symptom profile, only partially overlapping with that seen in young and middle-aged adults. For example, depressed older adults are less likely to report affective symptoms, which are heavily weighted in DSM criteria for MDD (Fiske et al., 2009). In fact, older adults generally report greater well-being (Carstensen et al., 2011). However, they are more likely to experience depression-related cognitive and somatic symptoms, including psychomotor retardation, as well as loss of interest in normal daily activities (Fiske et al., 2009). These symptoms may in turn contribute to subsyndromal depression, which may cause significant impairment in the quality of life of the elderly, without necessarily meeting DSM diagnostic criteria. Because depressive symptoms in the elderly are frequently distinct from those experienced by younger individuals and tend to be misattributed or overattributed to physical illness or bereavement, depression or subsyndromal depressive symptoms which impair quality of life in the elderly may be underdiagnosed and untreated (VanItallie, 2005). Moreover, early- and late-life onset depression likely represent distinct biological entities with different underlying pathophysiologies. Our findings conceivably tap into the pathophysiology of late-life onset depression, risk for or resilience to which may, however, be detectable at a younger age (Douillard-Guilloux et al., 2013).

Abnormally increased (Whalen et al., 2002; Victor et al., 2010; Yang et al., 2010) or decreased (Thomas et al., 2001; Lawrence et al., 2004; Wolfensberger et al., 2008) amygdala reactivity to threat or other emotionally salient stimuli have both been implicated in depression or risk thereof. The mixed nature of these findings may reflect distinct depression subtypes or pathways of risk. Heightened amygdala reactivity may be associated with increased sensitivity to environmental events and thus particularly predispose to depression in the context of stress (Caspi et al., 2010). In contrast, a relative blunting of amygdala response may reflect a reduced reactivity to environmental salience, which can be a risk factor for depression in its own right (Rottenberg et al., 2005; Bylsma et al., 2008). Our results suggest that the low-expressing major alleles at both rs7676614 and rs1391187/rs1909022 may confer risk for a subtype of MDD via the latter pathway. This notion is further strengthened by the fact that we observed no significant interaction between genotype and self-reported stress in predicting amygdala reactivity. Finally, this conceptualization is also consistent with a recent twin study which demonstrated that non-overlapping contributions of genetic and environmental factors to depression risk are associated with amygdala hypo- and hyper-reactivity, respectively (Wolfensberger et al., 2008). Taken together, these data suggest that *FREM3* functional variation may uniquely contribute to risk for a subtype of depression that is not precipitated by stress, but is rather a more proximal result of genetic influences. It should, however, be noted that effects of early life stress may emerge in a sample reporting higher and/or more variable levels of childhood trauma.

Importantly, *FREM3* is among the genes whose expression in the amygdala and ACC has previously been shown to decrease as a function of age (Glorioso et al., 2011). However, this prior work has used a cohort which may have been underpowered to detect changes in gene expression occurring across the entire adult lifespan. Here, using a cohort spanning the entire adult age range (16–96 years old), we extend these prior findings by reporting a robust and highly significant age-associate decrease in *FREM3* expression in Brodmann areas 11 and 47—regions broadly overlapping with the orbitofrontal cortex (OFC) as well as the ventrolateral prefrontal cortex (vlPFC), both of which have previously been implicated in depression pathophysiology and risk (Rogers et al., 2004; Stuhrmann et al., 2011).

Extensive prior work has shown that a shift toward a premature activation of age-related molecular pathways may increase risk for depression, among other neuropsychiatric disorders (Douillard-Guilloux et al., 2013; Sibille, 2013). In the current study, we report that the rs7676614 A allele, as indexed by the major allele of the proxy SNP rs6537170, is associated with a trend toward decreased *FREM3* expression in postmortem human brain tissue, independent of chronological age. This finding suggests that the A allele may predispose to depression via shifting *FREM3* expression to a pattern reminiscent of that seen in older age. Conversely, the minor G allele may be associated with relative resilience to the activation of these pathways and effectively contribute to a protective “slowing down” of normal age-related changes. Consistent with this conceptualization, one prior study has demonstrated a link between normal aging and the same *in vivo* phenotypic patterns we associated with the A allele (Tessitore et al., 2005). Specifically, using a BOLD fMRI paradigm similar to the one employed here, this study showed a relative reduction in amygdala reactivity and a concomitant slowing of face- but not shape-matching reaction times in older, relative to young, adults (Tessitore et al., 2005). Importantly, and in line with the current findings, this occurred in the absence of any differences in face or shape matching accuracy. Phenotypic convergence between older adults and carriers of the rs7676614 A allele is particularly remarkable in light of the fact that our sample consisted exclusively of young adults between the ages of 18 and 22.

The finding of slower reaction times in the face-matching condition in individuals with the rs7676614 A allele is further consistent with prior associations of the same allele and symptoms of psychomotor retardation (Shi et al., 2012). Furthermore, we found convergent effects of genotype on performance in the TMT, which is another index of psychomotor function. Importantly, we found a stronger genotype effect on performance in Trails B than in Trails A. Part A of the TMT is generally interpreted as a purer measure of motor function, while Part B taps into more complex visuomotor skills and task switching (Bowie and Harvey, 2006). The stronger genetic effect we observed in Part B, relative to Part A, suggests that rs7676614 may modulate higher-order cognitive processing of perceptual stimuli, rather than motor function alone. This notion is also consistent with the specificity of the rs7676614 effects on reaction times to the more perceptually and cognitively challenging face-matching condition, rather than the shape-matching condition of our amygdala reactivity paradigm.

While we found that the major allele of rs1391187/rs1909022, which is even more strongly linked to reduced *FREM3* expression, is similarly associated with reduced amygdala reactivity and slower TMT Part B performance, we found no effect of rs1391187/rs1909022 on RT in the Face or Shape matching condition or TMT Part A. It is possible that this SNP, due to its stronger impact on *FREM3*, may even more selectively impact complex, relative to simple, visuomotor tasks. The partial discrepancy in phenotypic association between rs7676614 and rs1391187/rs1909022 may also be attributed to differential LD patterns with additional variants, perhaps impacting the expression of neighboring genes.

Despite the effect of genotype on both neural reactivity and processing speed, we did not observe an association between TMT speed and RT and activity in either amygdala or BA11/47. Even though we investigated the effects of our SNPs of interest on gene expression only within regions of the OFC and vlPFC, *FREM3* is expressed in multiple brain regions (Hawrylycz et al., 2012). Thus, it is possible that the genetic effects on processing speed are primarily mediated through brain regions or systems not directly activated by our fMRI paradigm.

Importantly, we show that rs1909022, the SNP with a more robust effect on postmortem *FREM3* expression, is not only associated with differential amygdala response, but also modulates activity in regions of the OFC and vlPFC overlapping BA47. Specifically, we demonstrate that the low-expressing major allele is associated with blunted response to salient facial affect cues in BA47. Prior work has shown decreased OFC activity to negative (sad and angry) facial expressions in MDD (Lee et al., 2008), which may at least partially account for the deficits in emotion recognition and reactivity observed in currently depressed individuals (e.g., Persad and Polivy, 1993; Asthana et al., 1998). Other studies demonstrate OFC hyporeactivity may persist after disorder remission, but is remediated by acute administration of the antidepressant citalopram (Anderson et al., 2011). Additional research suggests that BA47 may be involved in selectively incorporating emotion into decision making (Beer et al., 2006). Specifically, activity in this region has been shown to increase when incorporating relevant negative emotional information into behavior, as well as when successfully inhibiting irrelevant negative information (Beer et al., 2006). Thus, hypoactivity in the same region may be associated with maladaptive use of emotion to guide behavior and constitute a risk factor for the development of depression. Furthermore, additional prior MRI research indicates that the structural and functional properties of the OFC may be particularly vulnerable to age-related decline, relative to other parts of the PFC (Lamar and Resnick, 2004; Resnick et al., 2007).

In addition to the effects of rs1909022 genotype on BA47 activity, we also observed a positive correlation between BA47 activity and extraversion. Prior work has demonstrated that higher extraversion may be protective against MDD, as well as psychopathology more broadly (Campbell-Sills et al., 2006). This, together with the fact that we associated the more common alleles at both loci with a relative blunting of neural reactivity, as well as slower processing speed, lends support to the conceptualization that the minor alleles may in fact be conferring relative resilience against depression- and age-related processes above and beyond what may be a normal “baseline” phenotype associated with the major alleles.

We further demonstrated that there was a positive correlation between amygdala response and BA47 activity, and, when taken together, these neural phenotypes both mediated the association between rs1909022 genotype and extraversion. The convergent effects of rs1909022 on both neural phenotypes, as well as their joint mediation effect, suggest that functional variation impacting *FREM3* expression likely modulates activity in multiple brain regions. Future work applying functional connectivity analytic approaches to task-based or resting state brain activity on the network level is likely to shed additional light on the full scope of these effects.

It should be noted that despite the fact that we observed a main effect of task, as well as an effect of rs1391187/rs1909022 genotype on gene expression, in both BA47 and BA11, we showed an effect of genotype on activity in BA47, but not BA11. Given the more ventral position of BA11, this region is more prone to signal dropout due to its proximity to tissue boundaries. Thus, it is possible that the most strongly activated and functionally relevant regions within BA11 were not adequately covered by our BOLD fMRI sequence. Future studies using imaging sequences optimized for coverage in the ventral OFC are likely to shed more light on the relative specificity of these and similar findings to lateral vs. more ventral regions of the OFC.

Critically, we found no direct link between genotype and current depressive or anxious symptomatology. However, it should be noted that our *in vivo* sample consisted of high-functioning young adults attending a competitive university. Thus, it is possible they are a particularly resilient population with relatively limited variability in depressive and anxious symptoms. Similarly, our postmortem sample contained individuals free of neuropsychiatric illness. In light of these sample characteristic, it might be reasonable to suggest that the genetic effects we observe on amygdala reactivity, perceptual processing speed, as well as gene expression, may reflect a latent vulnerability, which may only lead to disorder in the context of additional risk factors, including, but not limited to, increasing chronological age. Importantly, we did observe a link between the major allele of rs1391187/rs1909022 and the personality trait extraversion, partially mediated via differences in BA47 activity. Given prior links between extraversion and resilience to psychopathology independent of risk conferred by other personality traits (Campbell-Sills et al., 2006), our results may reflect a resilience phenotype associated with the high-expressing, rather than a vulnerability phenotype associated with the low-expressing *FREM3* alleles. Future studies utilizing prospective longitudinal designs, especially in cohorts enriched in elderly subjects and/or for MDD-related pathology, will be necessary to ascertain the validity of this notion.

The molecular mechanisms underlying the associations reported here have yet to be elucidated. The human *FREM3* gene codes for an extracellular matrix protein belonging to the same family as FRAS1, FREM2, and QBRICK/FREM1, which have been implicated in Fraser syndrome—a disorder characterized by severe congenital malformations in the development of the nose, ears, and throat, as well as mental retardation (van Haelst et al., 2007). However, no evidence has directly linked *FREM3* to Fraser syndrome and additional research in mouse models suggests the murine Frem3 gene shows distribution patterns distinct from those of Frem1 and Frem2 (Kiyozumi et al., 2007). As an extracellular matrix protein expressed in the brain, FREM3 is likely implicated in cell–cell interactions and maintaining the structural and functional integrity of nervous tissue. Thus, it is likely to be involved in neural development and function. Importantly, the extracellular matrix and its associated proteins have been shown to play an important role in brain aging (Morawski et al., 2014). Consistent with this, additional extracellular matrix proteins such as reelin also show strong, albeit less significant, age-related trajectories (Erraji-Benchekroun et al., 2005; Glorioso et al., 2011). Future studies directly manipulating the Frem3 gene in animal models would be needed to elucidate the role of this gene in normal and pathological brain function.

While both rs7676614 and rs1391187 were found to modulate *FREM3* gene expression in postmortem brain tissue, the precise molecular mechanisms via which these polymorphisms may exert their effects are unclear. Both rs7676614 and rs1391187, as well as their respective proxy SNPs rs6537170 and rs1909022, are intronic; thus, they may be involved in regulating alternative gene splicing. However, follow-up molecular genetic studies would be necessary to confirm this conjecture.

This study is not without its limitations. First, in order to ensure adequate statistical power and consistency with prior genetic association studies, we focused our analyses on non-Hispanic Caucasian individuals as they were the largest single ethnic group represented across samples. However, future studies should incorporate other ethnicities in order to more fully characterize the role of *FREM3* on depression risk. In addition, while we focused our *in vivo* analyses on the amygdala, we were unable to assess SNP effects on gene expression in that brain region. Prior work has demonstrated age-related decreases in *FREM3* expression in the amygdala and ACC (Glorioso et al., 2011), so it may be reasonable to assume that the cis-eQTL effect observed here also occur in the amygdala, although this will need to be tested directly. Indeed, drawing on prior work suggesting a general convergence of depression-associated molecular pathway dysregulation across diverse brain regions such as the ACC (Tripp et al., 2011), dorsolateral prefrontal cortex (dlPFC) (Sibille et al., 2011), and the amygdala (Sibille et al., 2009; Guilloux et al., 2012), we believe *FREM3* genetic variation would similarly impact expression patterns throughout the majority of cortical structures implicated in depression. Detailed molecular studies utilizing samples collected from different brain regions, ideally within the same individuals, would be necessary to test this conjecture.

In addition, while significant, the phenotypic differences we observed among genotype groups in our *in vivo* cohort were relatively small in size. This may be due to the fact that our sample consisted of high-functioning young adults with higher than average IQ, who were mostly free of current or past depressive symptomatology (Supplementary Table 1). Nonetheless, the fact that significant, albeit small, genetic differences emerged across multiple depression-relevant phenotypes even in this young and highly resilient sample may suggest that a potentially greater contribution of *FREM3* to depression risk could be unmasked in older and/or longitudinal samples, as well as those enriched for psychopathology. Our results should be interpreted with caution until confirmed by future research in such samples.

Last but not least, we focused on two polymorphic loci within a single gene, while our phenotypes of interest are known to be highly polygenic in nature. In this study, however, we did not set out to exhaustively explain phenotypic variability, but, rather, to illustrate how convergent results from multi-modal mechanistic follow-up studies and partial replications of suggestive GWAS hits may serve to improve biological knowledge of depression risk pathways. This knowledge can in turn be used to inform and guide future GWAS through phenotypic refinement or the enhancement of emergent variant prioritization techniques (e.g., Gagliano et al., 2014). Such synergy between large-scale genetic association studies and more targeted mechanistic approaches may not only offer better opportunities for the discovery of novel risk variants, but also promote an increasingly detailed delineation of biological pathways of risk, which may in turn open novel avenues for early individualized treatment and, possibly, prevention.

## Funding and disclosure

We thank Bartholomew Brigidi, Adam Gorka, Devin Jones, Annchen Knodt, and Spenser Radtke for their assistance in DNS data collection and analysis. The DNS is supported by Duke University and National Institute of Drug Abuse grant DA033369 (ARH). The gene expression work was supported by National Institute of Mental Health grants R01MH077159 and R01MH093723 (ES). The funding agencies had no role in the study design, data collection, and analysis, decision to publish, or preparation of the manuscript.

### Conflict of interest statement

The authors declare that the research was conducted in the absence of any commercial or financial relationships that could be construed as a potential conflict of interest.
