# Supplementary material for: FRAS1-related extracellular matrix 3 (FREM3) single-nucleotide polymorphism effects on gene expression, amygdala reactivity and perceptual processing speed: An accelerated aging pathway of depression risk
Source: Front Psychol. 2015 Sep 16;6:1377. doi: 10.3389/fpsyg.2015.01377 (PMC4584966; doi:10.3389/fpsyg.2015.01377)
Supplement: Supplementary file 1 [file Table1.DOCX]

**Supplementary Table 1.** Number of participants in our final sample meeting criteria for at least on DSM IV Axis I diagnosis.

| **DSM IV Axis I Diagnosis** | **n** |
| --- | --- |
| Agoraphobia (with or without history of Panic Disorder) | 3 |
| Alcohol Abuse | 17 |
| Alcohol Dependence | 12 |
| Bipolar disorder (past) | 1 |
| Generalized Anxiety Disorder | 2 |
| Major Depressive Disorder (current or past) | 8 |
| Obsessive Compulsive Disorder | 1 |
| Social Anxiety Disorder | 2 |
| Substance abuse (cannabis) | 3 |
| Substance dependence (cannabis) | 2 |
| Multiple diagnoses (with MDD comorbidity) | 7 |
| Multiple diagnoses (without MDD comorbidity) | 22 |
| **Total** | **80** |
