# Supplementary material for: FRAS1-related extracellular matrix 3 (FREM3) single-nucleotide polymorphism effects on gene expression, amygdala reactivity and perceptual processing speed: An accelerated aging pathway of depression risk
Source: Front Psychol. 2015 Sep 16;6:1377. doi: 10.3389/fpsyg.2015.01377 (PMC4584966; doi:10.3389/fpsyg.2015.01377)
Supplement: Supplementary file 3 [file DataSheet1.DOCX]

**FRAS1-related extracellular matrix 3 (*FREM3*) single-nucleotide polymorphism effects on gene expression, amygdala reactivity and perceptual processing speed**

Yuliya S. Nikolova,^1^ Swetha P. Iruku,^2^ Chien-Wei Lin^3^, Emily D. Conley,^4^ Rachel Puralewski,^5^ Beverly French,^5^ Ahmad R. Hariri,^2^ Etienne Sibille^1,5-6^

^1^Campbell Family Mental Health Research Institute of CAMH; Toronto, ON, Canada

^2^Laboratory of NeuroGenetics, Department of Psychology & Neuroscience, Duke University, Durham, NC, USA

^3^Department of Biostatistics, Graduate school of Public Health, University of Pittsburgh, PA, USA.

^4^23andMe, Mountain View, CA

^5^Department of Psychiatry, University of Pittsburgh, Pittsburgh, PA

^6^Departments of Psychiatry, and of Pharmacology and Toxicology, University of Toronto, Toronto, ON, Canada.

**Corresponding Author:**

Etienne Sibille, PhD

**Address:** Campbell Family Mental Health Research Institute at CAMH

250 College Street,

Toronto, ON, M5T 1R8, Canada

Etienne.Sibille@camh.ca

**Supplementary Methods**

*BOLD fMRI Data Acquisition*

Each participant was scanned using a research-dedicated GE MR750 3T scanner equipped with high-power high-duty-cycle 50-mT/m gradients at 200 T/m/s slew rate, and an eight-channel head coil for parallel imaging at high bandwidth up to 1MHz at the Duke-UNC Brain Imaging and Analysis Center. A semi-automated high-order shimming program was used to ensure global field homogeneity. A series of 34 interleaved axial functional slices aligned with the anterior commissure-posterior commissure (AC-PC) plane were acquired for full-brain coverage using an inverse-spiral pulse sequence to reduce susceptibility artifact (TR/TE/flip angle=2000 ms/30 ms/60; FOV=240 mm; 3.75×3.75×4 mm voxels; interslice skip=0). Four initial RF excitations were performed (and discarded) to achieve steady-state equilibrium. To allow for spatial registration of each participant’s data to a standard coordinate system, high-resolution three-dimensional structural images were acquired in 34 axial slices co-planar with the functional scans (TR/TE/flip angle=7.7 s/3.0 ms/12; voxel size=0.9×0.9×4 mm; FOV=240 mm, interslice skip=0).

*BOLD fMRI Data Preprocessing*

Images for each subject were realigned to the first volume in the time series to correct for head motion, spatially normalized into a standard stereotactic space (Montreal Neurological Institute template) using a 12- parameter affine model (final resolution of functional images=2 mm isotropic voxels), and smoothed to minimize noise and residual difference in gyral anatomy with a Gaussian filter, set at 6-mm full-width at half-maximum. Voxel-wise signal intensities were ratio normalized to the whole-brain global mean.

Variability in single-subject whole-brain functional volumes was determined using the Artifact Recognition Toolbox (http://www.nitrc.org/projects/artifact_detect). Individual whole-brain BOLD fMRI volumes meeting at least one of two criteria were flagged and regressed out when determining task-specific effects: 1) significant mean-volume signal intensity variation (i.e., within volume mean signal greater or less than 4 standard deviations of mean signal of all volumes in time series), and 2) individual volumes where scan-to-scan movement exceeded 2 mm translation or 2° rotation in any direction.

*BOLD fMRI Data Analysis*

The general linear model (GLM) of SPM8 (<http://www.fil.ion.ucl.ac.uk/spm>) was used to conduct fMRI data analyses. Following preprocessing, linear contrasts employing canonical hemodynamic response functions were used to estimate the effect of Face processing for the All Faces > Shapes contrast for each individual. Individual contrast images were then used in second-level random effects models accounting for scan-to-scan and participant-to-participant variability to determine mean condition-specific regional responses using one-sample t-tests. A statistical threshold of p<0.05, corrected across our amygdala and BA11/BA47 ROIs (as defined by the automatic anatomical labeling and Brodmann areas option in the Wake Forest University PickAtlas [1], respectively), and ≥10 contiguous voxels was applied. Notably and consistent with prior work [2; 3] , we extracted parameter estimates from our entire ROIs before performing any analyses involving additional variables.

*Postmortem samples*

Upon collection, coronal blocks are cut in ~2 cm blocks through the rostro-caudal extent of the brain and stored at –80C. The foremost part of the brain was collected for adequate pH (> 6.4) and for optimal RNA integrity (≥ 7.0) assessed by Agilent Bioanalyzer 2100 (Agilent Technologies, Palo Alto, CA). Orbitoventral prefrontal cortex (Broadman areas 11 and 47) are located within the 1st and 2nd prefrontal cortex blocks along the rostro-caudal axis. Samples containing all six cortical layers and excluding white matter were harvested from 2 consecutive 40 µm coronal sections as previously described [4], and immediately stored in TRIzol® Reagent (Invitrogen, Carlsbad, CA).

*Gene expression*

Total RNA was extracted from frozen samples using TRIzol® following manufacturer’s protocol. Briefly, brain samples were homogenized in TRIzol® and mixed with chloroform. After centrifugation at 10,000G for 18min, the mixture was separated into 3 phases: a lower red, phenol-chloroform phase, an interphase, and upper aqueous phase. Aqueous phase was carefully removed and transferred to a fresh tube. RNA was precipitated by mixing with the equal volume of 100% RNase-free ethanol and loaded into a column from RNeasy Micro Kit (Qiagen, Valencia, CA). RNA concentrations were determined by measuring absorbance at 260nm using Nanodrop ND-1000 Spectrophotometer (NanoDrop Technologies, Rockland, DE, USA). To confirm that RNA quality is high enough for large-scale gene expression study, 11 samples per brain region were randomly picked to measure RNA integrity by the Bioanalyzer 2100.

RNA samples were processed by the Gene Expression & Genotyping Core Facility at Case Western Reserve University. Briefly, cDNA was synthesized from 150ng of total RNA, by Ovation PicoSL WTA System V2 and labeled with Encore Biotin Module (both from NuGEN Technologies, San Carlos, CA). 2.5 µg of cDNA was hybridized on Affymetrix® Human Gene 1.1 ST arrays (Affymetrix, Santa Clara, CA), covering over 30,000 coding transcripts. Array hybridization, washing and staining were conducted on GeneTitan® (Affymetrix) according to the manufacturer’s protocol. ­

*Genetic variants*

Total DNA was extracted from fresh frozen brain samples with Qiagen DNA mini kit following manufacturer’s protocol. Shortly, ~25 mg of brain samples were homogenized and completely lysed with proteinase K at 56°C. 400 µg of RNase A was treated for 2 min at room temperature and inactivated by heating at 70°C for 10 min. DNA was precipitated with ethanol and applied to QIAamp Mini spin column. DNA concentrations were determined by measuring absorbance at 260nm using Nanodrop ND-1000 Spectrophotometer (NanoDrop Technologies, Rockland, DE, USA). The integrity of DNA was analyzed by 0.5% Agarose gel electrophoresis using 22 randomly picked samples. Utilizing SNP 6 Core reagent Kit from Affymetrix, 250ng of DNA was processed for hybridization onto Genome-Wide Human SNP array 6.0 featuring 909,622 SNPs (Affymetrix, Santa Clara, CA).

Genotype calls were generated using Affymetrix Genotyping Console version 4.1.3. For intensity quality control (QC), two samples (#1255 and #866) were removed by setting the Contract QC (CQC, which is the per sample QC test in the Affymetrix SNP 6.0 intensity array) threshold less than 0.4. Genotype calls were generated by Birdseed v2 algorithm, which uses EM algorithm to drive maximum likelihood fit of two dimensional Gaussian mixture model.

**References**

[1] J.A. Maldjian, P.J. Laurienti, R.A. Kraft, and J.H. Burdette, An automated method for neuroanatomic and cytoarchitectonic atlas-based interrogation of fMRI data sets. Neuroimage 19 (2003) 1233-9.

[2] J. Joeyen-Waldorf, Y.S. Nikolova, N. Edgar, C. Walsh, R. Kota, D.A. Lewis, R. Ferrell, S.B. Manuck, A.R. Hariri, and E. Sibille, Adenylate cyclase 7 is implicated in the biology of depression and modulation of affective neural circuitry. Biol. Psychiatry 71 (2012) 627-32.

[3] Y.S. Nikolova, E.K. Singhi, E.M. Drabant, and A.R. Hariri, Reward-related ventral striatum reactivity mediates gender-specific effects of a galanin remote enhancer haplotype on problem drinking. Genes Brain Behav 12 (2013) 516-24.

[4] D.W. Volk, M.C. Austin, J.N. Pierri, A.R. Sampson, and D.A. Lewis, Decreased glutamic acid decarboxylase67 messenger RNA expression in a subset of prefrontal cortical gamma-aminobutyric acid neurons in subjects with schizophrenia. Arch Gen Psychiatry 57 (2000) 237-45.
